# The Epidemiology of Lyme Borreliosis in Europe: An Updated Review on a Growing Public Health Issue

**DOI:** 10.1089/vbz.2022.0068

**Published:** 2023-04-12

**Authors:** James H. Stark, Andreas Pilz, Luis Jodar, Jennifer C. Moïsi

**Affiliations:** ^1^ Vaccines Medical Development, Scientific and Clinical Affairs, Pfizer Inc, Collegeville, Pennsylvania, USA.; ^2^ Pfizer Global Medical Affairs, Vaccines, Vienna, Austria.; ^3^ Pfizer Vaccines Medical Development, Scientific and Clinical Affairs, Paris, France.

In 1921, Arvid Afzelius published the first medical description of erythema migrans (EM) as a clinical entity. In multiple case reports from Sweden dating back to 1908, he postulated that EM resulted from an agent transmitted by the bite of the *Ixodes reduvius* tick. Subsequently, a link with neurological syndromes observed among individuals with a tick bite from the same geographic areas was hypothesized. In North America, the cutaneous lesion (with neurological sequelae) was first reported in 1968. A cluster of arthritis cases in the area of Lyme, Connecticut, followed in 1975 and was at the origin of the disease's name (Dammin, 1989).

Since the observation of EM in Europe and its recognition as Lyme borreliosis (LB), documentation of the disease's epidemiology has evolved substantially. Both Slovenia and the Czech Republic made LB notifiable in 1986. Subsequently, multiple countries developed public health surveillance systems for LB, yet no European-wide standardized public health surveillance system was established (Lindgren et al, 2006). In 2011, the European Union (EU) funded the European Union Concerted Action on LB (EUCALB) initiative to provide up-to-date information on LB-related activities throughout Europe for researchers, public health workers, and other relevant professional groups. EUCALB also established case definitions to help facilitate clearer and more uniform diagnosis, treatment, and reporting of LB across countries (Stanek et al, 2011). Most recently, the European Commission added Lyme neuroborreliosis to the list of diseases covered by epidemiological surveillance and authorized the European Centre for Disease Prevention and Control to monitor its EU-wide distribution (The Lancet Editorial Board, 2018). Despite these advancements in reporting and surveillance, the regional and local disease burden remains poorly understood.

This special issue of *Vector-Borne and Zoonotic Diseases* presents 10 up-to-date reviews of the epidemiology of LB in Europe over the past 15 years, complemented by granular analyses of electronic medical record (EMR) databases and public health surveillance data (laboratory and clinical diagnoses) from multiple European countries. Collectively, this special issue aims to collect all the available epidemiological knowledge of LB in Europe, highlight the growing disease burden problem, and call attention to areas where further research and harmonization are needed to better define the epidemiology of the disease and support preventive interventions.

The special issue begins with a comprehensive assessment of existing public health surveillance systems. Nagarajan et al summarizes characteristics of publicly available data for the 28 countries with LB public health surveillance, including statutory notification, case definitions and reported manifestations, years of data available, geographic coverage and granularity, and online dashboard availability. The assessment reveals the heterogenous nature of public health surveillance for LB in Europe, including the lack of appropriate surveillance, differences in quality of reporting, and different diagnostic methods employed, resulting in challenges for intercountry comparisons.

Building upon this public health surveillance assessment, Burn et al accesses surveillance data from 25 countries through published annual reports and online dashboards for data collected between 2005 and 2020. They found that national LB incidence was highest (>100 cases/100,000 population per year [PPY]) in Estonia, Lithuania, Slovenia, and Switzerland. Across the region, marked variation in disease incidence existed at the subnational level: many countries with low national incidence had areas of high endemicity. Overall, on average 128,888 cases are reported annually from these 25 countries with surveillance systems.

To further describe the incidence of LB within countries, a systematic literature review abstracted published national and subnational level incidence data for the period 2005–2020. Burn et al identified 61 unique articles that reported incidence data from 29 European countries including the United Kingdom. As seen in public health surveillance, case definitions varied dramatically across incidence studies, and fewer than one-quarter used the 2011 EUCALB definitions of LB manifestations. However, overall similar patterns were observed in the literature as in public health surveillance data, with higher incidence in four countries and substantial within-country variations across the continent.

The final summary assessment in this special issue investigates the seroprevalence of LB based on 61 articles identified from 21 European countries including the United Kingdom and reports variations in diagnostic testing strategy, national versus regional estimates, general population or risk group estimates, and by age and sex. In this review, seroprevalence was highest in countries in Western and Eastern Europe, but only one-quarter of the estimates were nationally representative of general population studies, pointing to the challenges of defining the true burden within many countries.

The next set of articles in this special issue presents country-specific analyses of individual-level data from EMRs, administrative or claims databases, and public health surveillance systems. Accessing these systems enabled the conduct of an in-depth analysis of local epidemiology and identify specific endemic localities. The first country-specific analysis, by Nuttens et al, reports and compares three sources of LB incidence data for France: the “Sentinelles” network (national surveillance of general practitioners), a separate general practitioners EMR data system, and the French national hospital discharge database. Collectively, this groundbreaking comprehensive evaluation of LB in France reveals high incidence in Limousin and the Northeastern regions and a disparity in recorded incidence between men and women, both in the hospital discharge database and in the general practitioner databases.

In the absence of national public health surveillance data for The Netherlands in the second analysis, Houben et al accessed the PHARMO general practitioner database to report LB, EM, and disseminated LB during the period 2015–2019. The analysis reveals an incidence rate ranging from 111 (95% confidence interval [CI]: 106–115) to 131 (95% CI: 126–136) per 100,000 person-years during this period, which is among the highest reported incidence rates in Europe and one that showed no indication of decline over time. The Eastern provinces of Drenthe and Overijssel (357 and 276 per 100,000 person-years, respectively) had the highest reported incidence; unfortunately, there were no data available from Western Germany to conduct a cross-border assessment.

In the third analysis, Skufca et al accessed German data from the online platform maintained by the Robert Koch Institute for the period 2016–2020. While the average annual LB incidence was 37.2/100,000 person-years at the national level, local disease risk was heterogeneous, ranging from 22.9 to 64.6/100,000 person-years among nine federal states, from 16.8 to 85.6/100,000 person-years among 19 regions within the federal states, and from 2.9 to 172.8/100,000 person-years among 158 counties.

In neighboring Poland, the incidence in the Western voivodeships was roughly equivalent to the incidence in the Eastern states of Germany, as is revealed in the analysis of the national public health surveillance system by Paradowska-Stankiewicz et al. This fourth analysis concludes that LB was endemic in all regions of Poland, with the highest incidence reported in the Eastern and Northeastern regions.

The final two articles in this special issue explore the disease burden in Finland. First, Skufca et al retrieved all reported LB cases (clinically diagnosed EM and microbiologically confirmed disseminated LB) for the period 2015–2020 from the online platforms the Register for Primary Health Care Visits (Avohilmo) and the National Infectious Diseases Register (NIDR), which are maintained by the National Institute for Health and Welfare. The average annual incidence was 99.6/100,000 residents (5478 cases), including the hyperendemic region of Ahvenanmaa, at 2473.9/100,000 residents. However, it is recognized that underascertainment of cases occurs with all public health surveillance systems.

To capture the full disease burden and estimate an underascertainment multiplier, Olsen et al used seroprevalence data and assumptions on the asymptomatic proportion and antibody duration to estimate the true incidence of LB in 2021. The analysis estimated that 19,653 LB cases occurred in 2021 (incidence of 526/100,000 per year) compared with 7,346 reported LB cases, resulting in a multiplier of 2.7 LB cases per case of LB reported by the National Institute for Health and Welfare in Finland. As a use case when the requisite data are available, this underascertainment method may be productive to estimate the true burden of LB.

Since the first case of EM in 1908 described by Arvid Afzelius, LB has been reported in nearly all countries within Europe. Numerous steps have been taken to foster robust, sustainable approaches to public health surveillance. However, questions remain about whether Lyme surveillance can accurately measure disease across countries given inconsistent implementation of standardized case definitions and reporting procedures. Even in countries reporting national incidence data, there is evidence of substantial subnational variation, suggesting that prevention efforts may need to rely on regional interventions, as well as national strategies, and raising the question of whether an incidence threshold to determine endemicity similar to the United States at 10 confirmed cases per 100,000 population can be applied to determine optimal preventive approaches. Predictive modeling using machine learning approaches may be useful here.

These questions notwithstanding, risk assessments have an integral role in prevention from a policy perspective. Understanding both distribution and quantity of Borrelia-infected ticks and prevalence of specific risk behaviors will drive aspects of perceived threats (*e.g.*, susceptibility), which can drive the intention to engage in prevention measures. In the past 15 years, LB burden has increased dramatically in endemic regions and has emerged into new areas. Countries should, therefore, continue to invest in high-quality disease surveillance, and individuals should maintain vigilance and recognize risk behaviors to prevent infection.
